# Development of an Automated Triage System for Longstanding Dizzy Patients Using Artificial Intelligence

**DOI:** 10.1002/oto2.70006

**Published:** 2024-09-27

**Authors:** Santiago Romero‐Brufau, Robert J. Macielak, Jeffrey P. Staab, Scott D.Z. Eggers, Colin L.W. Driscoll, Neil T. Shepard, Douglas J. Totten, Sabrina M. Albertson, Kalyan S. Pasupathy, Devin L. McCaslin

**Affiliations:** ^1^ Department of Otolaryngology–Head and Neck Surgery Mayo Clinic Rochester Minnesota USA; ^2^ Department of Biostatistics, Harvard T. H. Chan School of Public Health Harvard University Boston Massachusetts USA; ^3^ Department of Psychiatry Mayo Clinic Rochester Minnesota USA; ^4^ Department of Neurology Mayo Clinic Rochester Minnesota USA; ^5^ Department of Otolaryngology–Head and Neck Surgery Indiana University School of Medicine Indianapolis Indiana USA; ^6^ Department of Quantitative Health Sciences Mayo Clinic Rochester Minnesota USA; ^7^ Department of Biomedical and Health information Sciences University of Illinois‐Chicago Chicago Illinois USA; ^8^ Department of Otolaryngology‐Head and Neck Surgery University of Michigan Ann Arbor Michigan USA

**Keywords:** dizziness, Dizziness Handicap Inventory, functional vestibular disorder, psychiatric disorder, vestibular dysfunction

## Abstract

**Objective:**

To report the first steps of a project to automate and optimize scheduling of multidisciplinary consultations for patients with longstanding dizziness utilizing artificial intelligence.

**Study Design:**

Retrospective case review.

**Setting:**

Quaternary referral center.

**Methods:**

A previsit self‐report questionnaire was developed to query patients about their complaints of longstanding dizziness. We convened an expert panel of clinicians to review diagnostic outcomes for 98 patients and used a consensus approach to retrospectively determine what would have been the ideal appointments based on the patient's final diagnoses. These results were then compared retrospectively to the actual patient schedules. From these data, a machine learning algorithm was trained and validated to automate the triage process.

**Results:**

Compared with the ideal itineraries determined retrospectively with our expert panel, visits scheduled by the triage clinicians showed a mean concordance of 70%, and our machine learning algorithm triage showed a mean concordance of 79%.

**Conclusion:**

Manual triage by clinicians for dizzy patients is a time‐consuming and costly process. The formulated first‐generation automated triage algorithm achieved similar results to clinicians when triaging dizzy patients using data obtained directly from an online previsit questionnaire.

Dizziness and vertigo are extremely common symptoms, with estimations showing that 7% of women and 3% of men will require a medical consultation for vertigo at some point in their lifetime.^1^ Even more adults suffer dizziness that goes untreated, with 20% of the working population self‐reporting dizziness and over 50% of the elderly being affected as well.^2^, ^3^ Yet, causes of dizziness and vertigo, particularly when longstanding, are extremely difficult to diagnose. This diagnostic dilemma stems from the nonspecific, cross‐disciplinary nature of dizziness as different etiologies may present in a similar manner while requiring substantially different diagnostic evaluation and treatment strategies.

Management of the various causes of dizziness spans the domains of several health care specialties. This creates a substantial challenge for primary care providers from both a diagnosis and a referral standpoint.^4^ Consequently, there exists broad variability and inconsistency in the management, care, and referral pathways.^5^, ^6^ Many patients attend inappropriate visits and undergo unnecessary tests attempting to diagnose and treat their condition.^7^, ^8^ Furthermore, nearly 23% of patients present to an emergency department as their initial encounter for these symptoms.^9^ Often no solution is found, as up to 86% of patients receive the diagnosis of “unspecified dizziness.”^10^ This is further magnified by a significant associated financial cost, with the annual cost of dizziness evaluations now exceeding $4 billion a year to US emergency departments alone.^11^ Hence, substantial time and money are invested by patients, providers, and payers, often with no resolution of symptoms or formal diagnosis. To combat these issues, an effective triage system could reduce needless physician visits for patients while producing considerable cost savings.^9^

At tertiary and quaternary levels of care, the process of determining which specialists are best suited to address patients' dizziness is just as challenging. As no one specialty can address every cause of dizziness, patients with select sets of symptoms are commonly routed to clinicians deemed most appropriate for their constellations of complaints. However, referrals to different disciplines may not necessarily translate into improved outcomes because training and experience treating dizziness widely varies. When evaluating patients with dizziness, particularly longstanding dizziness, comprehensive documentation of the patient's symptoms is needed to develop a hypothesis regarding the potential diagnosis. Ideally, symptom reports and initial differential diagnoses would lead to appointments with appropriate specialists. Multiple studies have investigated the capability and limitations of using various tools to *diagnose* the causes of dizziness, including self‐reported questionnaires, decision‐making guides, and machine learning algorithms.^12^, ^13^, ^14^, ^15^, ^16^, ^17^, ^18^, ^19^ Overall, they show promise but are limited in scope and accuracy. A singular study by Friedland et al sought to use such tools for a working diagnosis and recommendations on initial management options, aiming to optimize this process and help patients receive the care they need.^20^

Over the past 2 decades, this institution has worked to pioneer a coordinated multidisciplinary approach to diagnosing and treating patients with balance disorders, with a particular focus on patients with longstanding dizziness, or dizziness that is persistent or episodic and has lasted for at least 3 months.^21^, ^22^ Currently, prior to patients' initial visits, a symptom self‐report questionnaire is completed. The results of that report and outside medical records are reviewed by an expert clinician who recommends a set of appointments necessary to diagnose and treat the patients based on an initial differential diagnosis. This method has been shown to be effective in improving patient outcomes^21^, ^22^; however, this process requires substantial effort and can still result in inappropriate or unnecessary appointments. The purpose of the present study was to automate and optimize scheduling of multidisciplinary consultations for patients with dizziness through the use of a proprietary machine learning algorithm.

## Methods

### Data Sets

The study was approved by Mayo Clinic's Institutional Review Board (IRB# 18‐000088). Included patients were referred from outside the institution and received the standard medical care from the authors' multidisciplinary program with specialty and subspecialty referrals managed by a traditional manual system. This manual method of triage performed as part of normal clinical practice consisted of a review of a questionnaire and outside records completed by an audiologist with specific training in balance disorders. The questionnaire consists of 162 items that queried the duration of the patients' symptoms, previous appointments before requesting care at the authors' institution, frequency of balance/dizziness symptoms, and other symptoms such as hearing change or headache. Notably, most patients do not see all items depending on their response. Diagnostic information used for reporting purposes was captured from the medical record following completion of all recommended consultations.

The initial step of model development was variable selection. This was performed on a data set of patients who had completed the questionnaire (N = 225), and it included the questionnaire results as predictors and the result of the clinical prospective review as the ground truth. The purpose of this step was simply to inform us what questions would be informative, and they are listed in Table 1. This clinical prospective review was the triage performed as part of routine clinical practice, where appointments were selected by a single reviewer.

**Table 1:** Contributing Questions for Model Formulation

QuestIon/statement verbiage
Do you ever have symptoms occur when you are sitting, standing or lying completely still, NOT having just moved and NOT watching anything that is moving?
I have a documented hearing loss.
I have ringing or noise that I hear.
I have a feeling of fullness or pressure in my ear(s).
Are your symptoms made worse by any of the following?: Loud sounds, lifting/straining, or coughing/sneezing/nose blowing.
Have you ever had surgery on either ear?
Sounds are too loud.
I hear my own voice louder than other voices.
I get dizzy when I strain to lift something heavy.
I get dizzy when I have a bowel movement.
Loud sounds make me dizzy.
I get dizzy when I sneeze.
When I cough I get dizzy.
Do you hear body sounds (eyes move or blinking)?
I have pain in my ear(s).
Have headaches been a significant problem within the past 6 months?
Have you ever experienced a temporary change in your vision, such as jagged lines, color spots, or lightning bolts in your vision?
Please check all of the following that you have experienced: Increased sensitivity to light during a headache, increased sensitivity to sound during a headache, increased sensitivity to odors during a headache, headaches associated with your problems of dizziness or imbalance.
How long on average does a typical spell last?
How frequently do your spells occur?
Are your symptoms made worse by any of the following?: Automobile rides, reading, stress or nerves, supermarket aisles/malls/tunnels, windshield wipers, restaurants or movie theaters.
Have you ever felt a sensation of rocking or swaying?
On the PHQ‐4, is your Anxiety subscale ≥2?
On the PHQ‐4, is your Depression subscale ≥2?
Have you ever been diagnosed with a stroke?
Do you have abnormal findings on a computed tomography (CT) scan of the head?
Do you have abnormal findings on magnetic resonance imaging of the head with contrast?
Do you have abnormal findings on magnetic resonance imaging of the head without contrast?
Are your symptoms with you 24 hours per day, never stopping?
Do you feel off‐balance when standing or walking?
Have you experienced unexplained falls?
Have you been diagnosed in the past with ongoing numbness or tingling?

Abbreviation: PHQ‐4, Patient Health Questionnaire‐4.

To develop the algorithm's development data set, members of a multidisciplinary team performed a retrospective analysis of the diagnoses and scheduled appointments for a randomly selected cohort of patients (N = 98) that were previously evaluated, diagnosed, and treated in the clinic. The team having the knowledge of the final diagnoses from the initial consultations, created “ideal” itineraries after extensive discussion with a unified recommendation. This expert panel was comprised of 4 to 5 members and included at least the following experts: a neurologist with specific training in neuro‐otology, a psychiatrist with specific expertise in dizziness, a board‐certified neurotologist, and at least 1 audiologist with specific training in dizziness disorders. To allow for a gold‐standard triage decision, all necessary information was available, and the multidisciplinary team was not blinded to this information. Also, instances where appointments were necessary to rule out a diagnosis even if unlikely were taken into account and added accordingly.

The group used an N − 1 approach, that is, consensus was considered reached when the number of dissenting opinions was not more than 1, to reach consensus; however, within this study, all appointment decisions were ultimately made with a complete consensus despite this approach. Diagnoses that were considered in the development of the best itinerary—defined as an itinerary that was comprehensive in determining a diagnosis and management—to address the patients' balance complaints were abstracted from the reports of each clinical consultation in accordance with the International Classification of Vestibular Disorders, International Classification of Diseases, 10th edition, and Diagnostic and Statistical Manual of Mental Disorders, fifth edition. The primary specialty appointments that were available for consideration were otolaryngology (medical and surgical), neurology (general, headache specialist, and otoneurology), and psychiatry/psychology (general psychiatry and vestibular psychiatry). In addition, the panel could determine which additional tests or additional specialty consultations, if any, would be appropriate. Prior imaging studies were available prior to arrival for all cases. The multidisciplinary expert panel consensus of necessary appointments was then compared to the actual appointment set that had been selected using the historical single‐clinician triage system.

Our model validation data set consisted of an additional 52 prospective patients, who requested an appointment after the model had been developed, that were evaluated by the expert panel using a similar approach to the one described for the model development data set.

### Algorithm Development

The triaging algorithm was developed with a mixed approach using statistical modeling and clinical expertise. One model was developed for each appointment category; for each model, we used logistic regression models and gradient‐boosting models, with the questions as the predictors and each appointment type as the response. We analyzed the model parameters as well as the relative importance of each question in the gradient boosting model to determine which variables were informative in the scheduled appointment. As noted, 98 subjects were randomly selected to go through the group consensus for what appointments they should have received. These patients were not included in the original model development data, and consensus results were noted for all of these patients.

We first ran 2 sets of models (gradient boosted tree models [GBM] and linear models) using the prospective patient questionnaire data (N = 225) as predictors and the clinical triage decision as the outcome. A separate model was built for each appointment type. A subset of predictors was selected using the variable importance values from the GBM model, the coefficients from the linear model, and expert selection. From that set of a predictors, a decision tree was built that combined the information obtained from the GBM and linear models, and a set of dependencies obtained from the experts. For example, if a patient needed an appointment at a neurovestibular subspecialist, an additional appointment with a general neurologist was not needed. The decision tree also ensured that all patients received an appointment with at least 1 specialty. The decision tree was then adjusted based on expected appointment availability by running the model through the historical data set and adjusting the threshold for different appointments based on how many patients would be referred to that specialist. This ensured that specialties with a lower availability of appointments received patients that could benefit most from an appointment and allows the model to consider availability of appointments in the different clinical departments. Furthermore, the sequence of appointments was taken into account with the sequence largely staying the same for a specific set of recommended appointments. The algorithm was developed using R statistical software version 3.4.1 and was implemented using a docker container.

### Algorithm Validation

The performance of the algorithm's predictions was then evaluated by comparing the appointment itineraries selected by the algorithm to the consensus decision of the expert panel of clinicians on the validation data set of 52 prospective patient cases. Specifically, each appointment type that the algorithm selected for each patient was reviewed and matched against the opinion of the expert panel (Supplemental File S1, available online).

### Statistical Analysis

In addition to reporting the descriptive data from this sample, the level of agreement between our current manual system of questionnaire‐based triage and a multidisciplinary expert team was calculated using Cohen's *κ* coefficient for “Algorithm Development.” For “Algorithm Validation,” Cohen's *κ* coefficient was also used to determine the magnitude of agreement between the model's assignment of appointments and the expert panel review. The Cohen's *κ* statistic accounts for the percentages of agreement that would be expected by chance. Statistical interpretation was based on Landis and Koch criteria.^23^ Accordingly, interpretation of values between 0.0 and 0.2 to indicated slight agreement, 0.21 and 0.40 to indicated fair agreement, 0.41 and 0.60 to indicate moderate agreement, 0.61 and 0.80 to indicate substantial agreement, and 0.81 and 1.0 to indicate almost perfect/perfect agreement. All data were analyzed with version 9.4 of the SAS software package (SAS Institute).

## Results

### Algorithm Development

**Figure 1:**
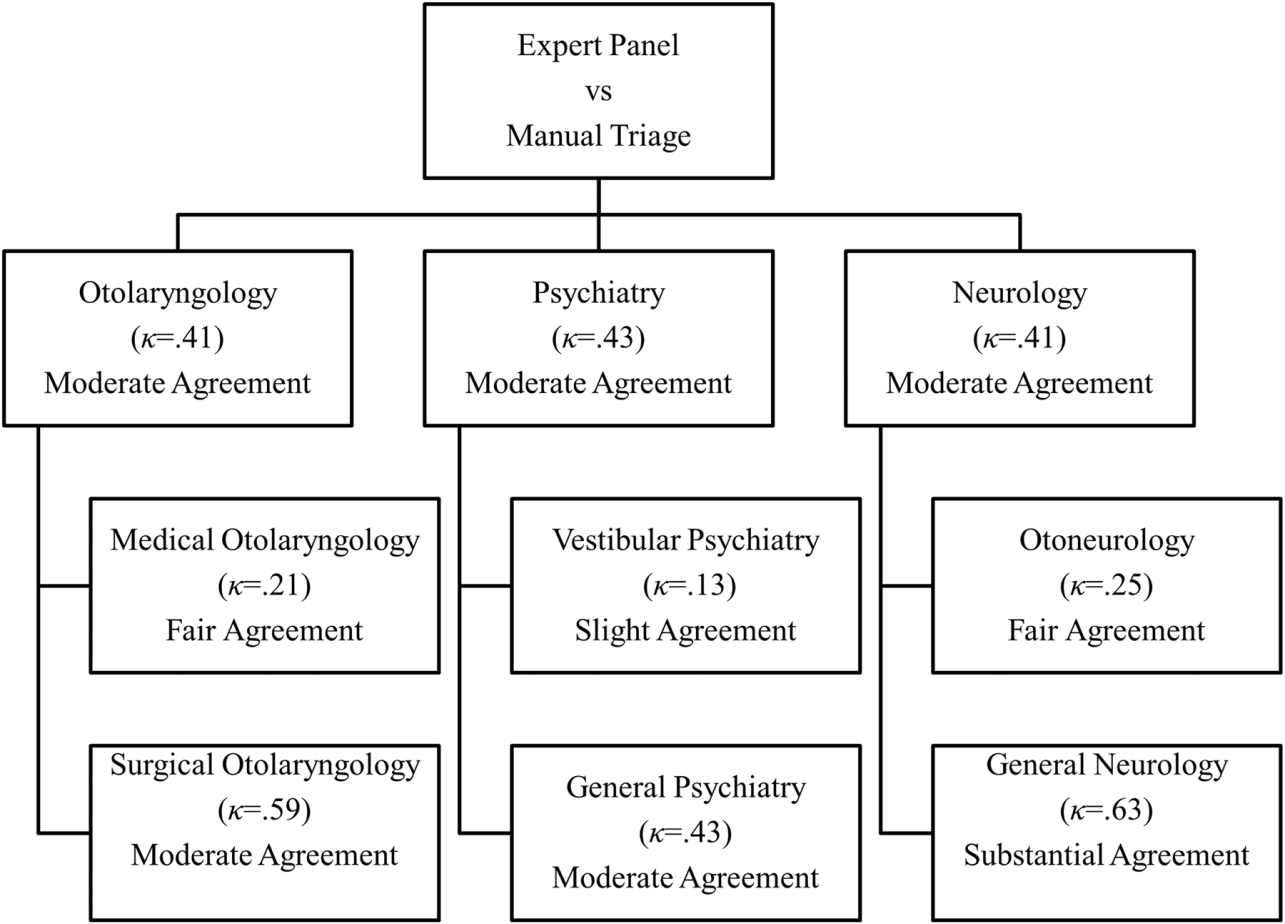
Cohen's *κ* coefficients showing the level of agreement for selected appointment itineraries between the manual triage system and expert panel for each of the disciplines included in the care pathway.

The initial set of training data for the algorithm consisted of previsit self‐report questionnaires from 98 randomly selected patients evaluated between February 2016 and May 2018 (Table 2). The concordance between the historical manual triage approach and the expert team adjudication for the primary disciplines of otolaryngology, neurology, and psychiatry were 70% (95% confidence interval [CI]: 60‐79), 69% (95% CI: 59‐78), and 74 (95% CI: 65‐83), respectively. There was a moderate level of agreement for all 3 groups (Figure 1).

**Table 2:** Patient Demographics and Multidisciplinary Diagnoses Contributing to Vestibular Symptoms in Algorithm Development and Algorithm Validation Data Sets

	Expert panel algorithm development (N = 98)	Algorithm validation (N = 52)
Demographics		
Age, y (mean ± SD)	56 ± 12	55 ± 14
Sex F/M (mean%)	65/33 (34%)	33/19 (35%)
Diagnoses^a^		
Peripheral vestibular disorders		
Acute vestibular syndrome (by history)^b^	8	1
Benign paroxysmal positional vertigo (by history)^c^	9	
Benign paroxysmal positional vertigo (active)^d^	1	1
Cervical vertigo (post‐operative)	1	
Meniere's disease	14	12
Semicircular canal dehiscence syndrome	2	1
Unilateral vestibulopathy^e^	9	10
Vestibular schwannoma	1	1
Bilateral vestibular hypofunction	1	1
Central vestibular disorders		
Migraine/Headache disorder^f^	15	10
Vestibular migraine^g^	14	6
Traumatic brain injury/whiplash	4	
Central vestibular deficit, unspecified	2	2
Central oculomotor deficit	2	
Hearing loss		
Sensorineural hearing loss	49	31
Conductive hearing loss	3	2
Other structural diagnoses		
Spinal disease (cervical, lumbar)	2	
Autonomic disorders^h^	6	1
Stroke	2	3
Functional vestibular disorders		
Mal de debarquement syndrome	3	1
Persistent postural‐perceptual dizziness	41	16
Psychiatric disorders		
Generalized anxiety disorder	21	3
Panic disorder ± agoraphobia	10	2
Specific phobia (dizziness or falling)	7	
Other anxiety disorder	5	
Major depressive disorder	9	1
Other depressive disorder	3	
Bipolar disorder	1	
Posttraumatic stress disorder	4	
Somatic symptom disorder	4	

Abbreviations: F, female; M, male.

^a^ Totals are greater than the number of patients in each category because of multiple comorbid diagnoses.

^b^ History of an acute episode of spontaneous onset vertigo lasting for days to weeks without evidence of residual structural deficits.

^c^ History consistent with benign paroxysmal positional vertigo, but negative positional testing at the time of evaluation.

^d^ Active benign paroxysmal positional vertigo on physical examination.

^e^ Presence of unilateral peripheral vestibulopathy on laboratory testing, Meniere's disease, or canal dehiscence.

^f^ Diagnosis of any migraine or other headache disorder, excluding vestibular migraine.

^g^ Diagnosis of vestibular migraine or probable vestibular migraine.

^h^ Postural orthostatic tachycardia syndrome, orthostatic intolerance, neurocardiogenic syncope.

The level of agreement for all subspecialties between the expert team triage and the manual triage is summarized in Table 3. The mean concordance between the expert team triage and the manual triage across all subspecialties was 70%.

**Table 3:** Concordance Values From Algorithm Training and Validation for All Available Subspecialties

Subspecialty	Expert panel training, % (95% CI)	Algorithm validation, % (95% CI)
Medical otolaryngology	69 (59‐78)	69 (55‐81)
Surgical otolaryngology	85 (76‐91)	87 (74‐94)
General neurology	62 (52‐72)	83 (69‐91)
Headache neurology	99 (94‐100)	90 (78‐96)
Otoneurology	67 (57‐76)	85 (78‐96)
General psychiatry	53 (43‐63)	63 (49‐76)
Vestibular psychiatry	57 (47‐67)	79 (65‐88)

Abbreviation: CI, confidence interval.

### Algorithm Validation

**Figure 2:**
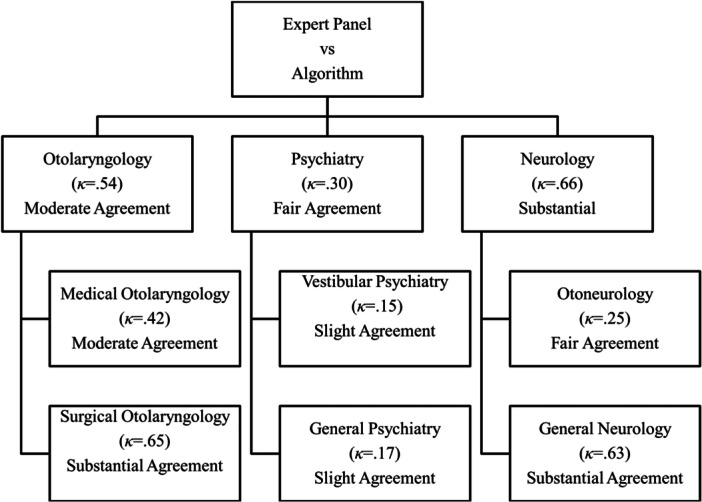
Cohen's *κ* coefficients showing the level of agreement for selected appointment itineraries between the algorithm and the expert panel review for each of the disciplines included in the care pathway.

The algorithm validation data was composed of 52 patients (Table 2). The concordance between the algorithm triage and the expert team adjudication for the primary disciplines of otolaryngology, neurology, and psychiatry were 83% (95% CI: 69‐91), 83% (95% CI: 69‐91), and 65% (95% CI: 51‐78), respectively. There was a moderate of agreement for all groups (Figure 2).

The level of agreement for all subspecialties between the algorithm triage and the expert panel review is summarized in Table 3. The mean concordance between the algorithm triage and the expert team triage across all subspecialties was 79%.

## Discussion

The current study was undertaken to develop and validate a system capable of automating the triage of patients with longstanding dizziness. The first part of this investigation was undertaken to determine which questions on a patient questionnaire were predictive of certain subspecialties and use this information to train an algorithm. The second part of this investigation sought to subsequently validate this algorithm's triage success through comparison with an expert panel of clinicians and a prospective review.

As evidenced by the obtained results, an algorithm was successfully formulated through this process, and it was found to have a moderate level of agreement and mean concordance of 79% with the expert triage, superior to the concordance achieved by the original clinical triage decision. This novel process allowed for excellent overall results with limited required manual input, serving as a starting point for further implementation of an automated triage process. As additional data points are provided, the algorithm will continue to be further refined with better anticipated results.

Literature in this realm has been limited, with most of studies designed to provide a formal working diagnosis rather than a suggestion regarding the most appropriate specialist/s that would be capable of providing effective treatment.^19^ The most comparable investigation at present is the work by Friedland et al, which sought to compare symptoms with diagnoses for the goal of triage.^20^ The investigators adapted the questionnaire used in the current study and evaluated its accuracy in classifying a number of vestibular disorders. Final diagnoses were matched statistically to specific items from the questionnaire. Results showed the questionnaire could identify benign paroxysmal positional vertigo (BPPV) with a sensitivity of 79%, Meniere's disease with a sensitivity of 81%, and vestibular migraine with a sensitivity of 76%.

The significance of the current study relates to the novel approach taken in order to arrive at the fastest and most accurate diagnosis for patients with longstanding dizziness. Rather than attempting to assign a formal diagnosis using only symptoms, the design of the current project centers on routing patients to the most appropriate specialties based on these symptoms. This approach enables the treating specialist to continue refining the differential diagnosis through incorporation of additional information derived from the physical examination, imaging, or laboratory testing before rendering a formal diagnosis and recommending treatment. Furthermore, due to the effect of additional symptoms associated with medical comorbidities, a questionnaire may not clearly detect any definitive diagnosis and likely lacks the comprehensive information that may be needed to make a diagnosis. In many instances, deciding on the causes of a patients' symptoms following an appointment with the treating professional allows for a more informed diagnosis and reduces the chance of unnecessary appointments and testing.

Further, a number of the clinical tools developed to assign possible diagnoses focus only on identifying common neuro‐otologic syndromes. In order to develop these items, symptoms are often extracted from published criteria (eg, Barany Society's International Classification of Vestibular Disorders^24^), and a diagnosis is assigned to the patient when a certain set or pattern of questions is endorsed. However, this could be a limiting factor for such an endeavor. The consensus report on BPPV provides a telling example.^25^ The 13‐page report states that in order to deliver an accurate diagnosis for BPPV, the semicircular canal that is affected must be identified as well as a differentiation between canalolithiasis and cupulolithiais. Additionally, there are numerous caveats presented, for example, differentiating positional vertigo from orthostatic symptoms. Considering these points, it is an extremely challenging task to make such a diagnosis using a symptom‐based questionnaire. In summary, although the development of questionnaires to provide an initial working diagnosis based on a set of reported symptoms is a valuable task, in cases where patients are suffering from the complex diagnoses associated with dizziness, they may have limited clinical utility.

The next phase of practice improvement involving the current triage algorithm will be multifaceted. It is the authors' intention to: (1) develop an online patient‐facing portal that will administer the questionnaire described in the current study and house the data; (2) adjust the machine learning model based on the learnings from this study including combining the algorithm's automation with feedback from the expert clinicians; (3) assess the accuracy of this model in straightforward versus more complex diagnoses; and (4) develop a provider‐facing interface displaying the recommendations and measure its useful and accuracy in a real world setting.

A limitation of this investigation was that the patient sample was composed entirely of patients seen in the author' multidisciplinary longstanding dizziness clinic, which resulted in a disproportional number of patients with atypical dizziness. This may limit the generalizability of our algorithm. Additionally, there is potential for suspected diagnoses and thus triage results to change with delays in evaluation, as diagnoses such as persistent postural‐perceptual dizziness tend to develop in a delayed fashion after vestibular dysfunction of different etiologies. This nuanced point was not incorporated into this initial feasibility study. Further, the retrospective nature of the study could have limited the potential accuracy of the algorithm, which could improve when applied prospectively in a real‐world setting. A particular strength of the study was that all specialists and subspecialists involved in the care of dizziness were available. The comprehensive nature of the multidisciplinary team allowed specific diagnoses in multiple areas to be assigned to the patients in the sample; however, implementation of such algorithms at centers without subspecialty care would certainly necessitate addition of a recommendation for referral to a tertiary center offering such service. Furthermore, acknowledging this limitation in applicability to all centers, the data from this and future studies may inform practice by disseminating the most effective treatment options and recommendations to the broader health care community, allowing for more efficient referral even if this technology is not utilized.

## Conclusion

Manual triage by clinicians is a time‐consuming and costly process. This first‐generation automated triage algorithm achieved similar results to clinician experts, using data obtained directly from an online previsit questionnaire, potentially increasing efficiency in scheduling patients. Future advances in the triage algorithm will improve concordance with ideal itineraries and offer an opportunity to provide more cost‐effective patient care.

## Author Contributions

**Santiago Romero‐Brufau**, study design, data collection, analysis, manuscript drafting and revision, final approval, agreement to be accountable for all aspects of the work; **Robert J. Macielak**, analysis, manuscript drafting and revision, final approval, agreement to be accountable for all aspects of the work; **Jeffrey P. Staab**, study design, data collection, manuscript revision, final approval, agreement to be accountable for all aspects of the work; **Scott D.Z. Eggers**, study design, data collection, manuscript revision, final approval, agreement to be accountable for all aspects of the work; **Colin L.W. Driscoll**, study design, data collection, manuscript revision, final approval, agreement to be accountable for all aspects of the work; **Neil T. Shepard**, study design, data collection, manuscript revision, final approval, agreement to be accountable for all aspects of the work; **Douglas J. Totten**, data collection, manuscript revision, final approval, agreement to be accountable for all aspects of the work; **Sabrina M. Albertson**, analysis, manuscript revision, final approval, agreement to be accountable for all aspects of the work; **Kalyan S. Pasupathy**, study design, data collection, manuscript revision, final approval, agreement to be accountable for all aspects of the work; **Devin L. McCaslin**, study design, data collection, analysis, manuscript drafting and revision, final approval, agreement to be accountable for all aspects of the work.

## Disclosures

### Competing interests

The model described in the manuscript was licensed by Mayo Clinic to a commercial third party, and J.P.S., S.D.Z.E., N.T.S., C.L.W.D., D.L.M., S.M.A., K.S.P., and S.R.‐B. are listed as inventors and received royalties as part of the license agreement. None of the co‐authors have received any income from the company in the last 24 months.

### Funding source

No funding or other support was required for this study.

## Supporting information

Supporting information.
