# Supplementary material for: Development of an Automated Triage System for Longstanding Dizzy Patients Using Artificial Intelligence
Source: OTO Open. 2024 Sep 27;8(3):e70006. doi: 10.1002/oto2.70006 (PMC11427795; doi:10.1002/oto2.70006)
Supplement: Supplementary file 1 — Supporting information. [file OTO2-8-e70006-s001.docx]

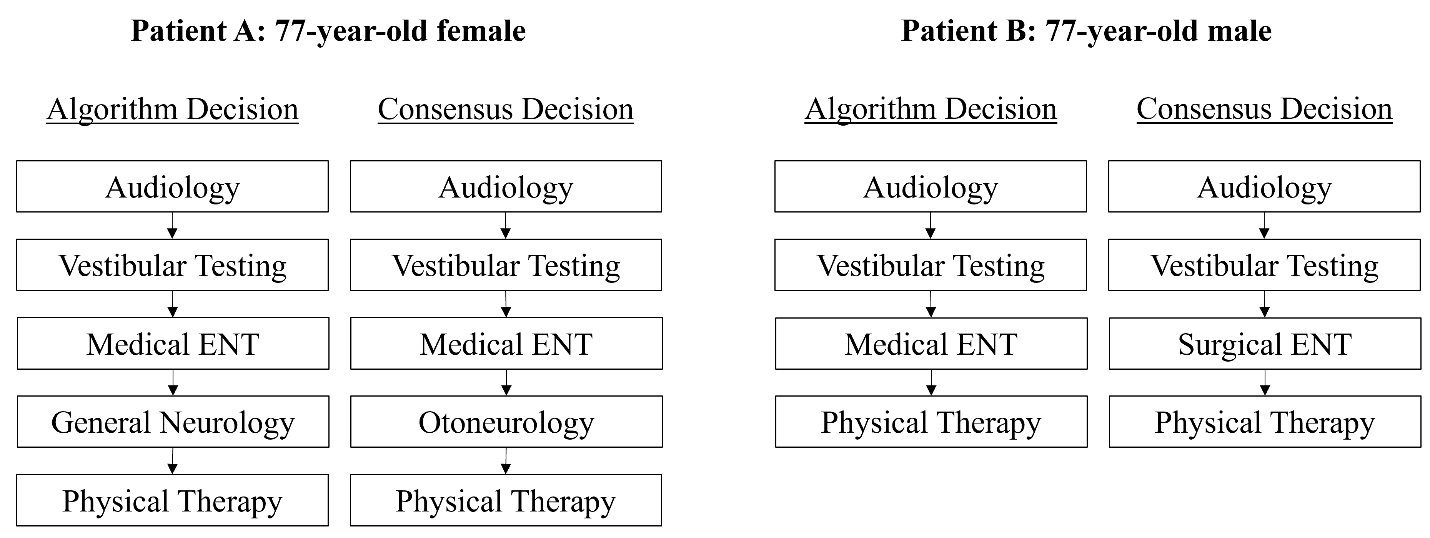


**Supplemental Information 1.** Examples of patient itineraries based on consensus triage versus algorithm triage exemplifying the validation of the algorithm.
